# Amlodipine inhibits Synaptotagmin-4’s oncogenic activity on gastric cancer proliferation by targeting calcium signaling

**DOI:** 10.1007/s10142-024-01345-8

**Published:** 2024-04-18

**Authors:** Wen Huang, Shuo Yang, Minying Deng, Rongkui Luo, Huaiyu Liang, Yanyan Shen, Biyu Yang, Chen Xu, Yingyong Hou

**Affiliations:** 1 Department of Pathology, Zhongshan Hospital, Fudan University, 180 Fenglin Road, Xuhui District, Shanghai, 200032 China; 2 Department of Orthopaedics, People’s Hospital of Tongzhou Bay Demonstration Zone, Nantong, Jiangsu China; 3 Department of Orthopaedics, Nantong First People’s Hospital, Affiliated Hospital 2 of Nantong University, Nantong, Jiangsu China; 4 Shanghai Institute of Materia Medica, Chinese Academy of Sciences, Shanghai, 201203 China

**Keywords:** Gastric cancer, synaptotagmin-4, Calcium ion channel, Mitogen-activated protein kinase signaling pathway, Amlodipine

## Abstract

Background: Gastric cancer (GC) remains a leading cause of cancer mortality globally. Synaptotagmin-4 (SYT4), a calcium-sensing synaptic vesicle protein, has been implicated in the oncogenesis of diverse malignancies. Purpose: This study delineates the role of SYT4 in modulating clinical outcomes and biological behaviors in GC. Methods: We evaluated SYT4 expression in GC specimens using bioinformatics analyses and immunohistochemistry. Functional assays included CCK8 proliferation tests, apoptosis assays via flow cytometry, confocal calcium imaging, and xenograft models. Western blotting elucidated MAPK pathway involvement. Additionally, we investigated the impact of the calcium channel blocker amlodipine on cellular dynamics and MAPK pathway activity. Results: SYT4 was higher in GC tissues, and the elevated SYT4 was significantly correlated with adverse prognosis. Both univariate and multivariate analyses confirmed SYT4 as an independent prognostic indicator for GC. Functionally, SYT4 promoted tumorigenesis by fostering cellular proliferation, inhibiting apoptosis, and enhancing intracellular Ca^2+^ influx, predominantly via MAPK pathway activation. Amlodipine pre-treatment attenuated SYT4-driven cell growth and potentiated apoptosis, corroborated by in vivo xenograft assessments. These effects were attributed to MAPK pathway suppression by amlodipine. Conclusion: SYT4 emerges as a potential prognostic biomarker and a pro-oncogenic mediator in GC through a Ca^2+^-dependent MAPK mechanism. Amlodipine demonstrates significant antitumor effects against SYT4-driven GC, positing its therapeutic promise. This study underscores the imperative of targeting calcium signaling in GC treatment strategies.

**Supplementary Information:**

The online version contains supplementary material available at 10.1007/s10142-024-01345-8.

## Introduction

Gastric cancer (GC) is a leading cause of cancer mortality worldwide, attributed to its genetic complexity and aggressive behavior (.Gao et al. 2015). According to the International Agency for Research on Cancer’s 2020 Cancer Burden data, GC accounts for the sixth in incidence and the third in mortality among all malignancies(.Sung et al. 2021). The 2016 cancer statistics estimated that approximately 397,000 new cases of GC were diagnosed in China each year, with about 289,000 deaths, ranking the third in both incidence and mortality (.Zheng et al. 2022). The disease’s heterogeneity and the advanced stage at which it is typically diagnosed contribute to a persistently low 5-year survival rate (.Ji et al. 2004), underscoring the urgent need for novel prognostic biomarkers and more effective therapeutic strategies.

Recent studies highlight the significance of ion channels, particularly those involved in Ca^2+^ signaling, as novel therapeutic targets in cancer (.Shiozaki et al. 2021). Ca^2+^ signaling is crucial in various cell death pathways (.Kokoska et al. 1998), warranting further exploration of its role in GC progression.

Synaptotagmin-4 (SYT4) is a member of a family of membrane proteins that act as calcium sensors, with 17 known isoforms (.Spaiardi et al. 2020). It functions as a postsynaptic Ca^2+^ sensor, triggering retrograde signals that enhance presynaptic function via the cAMP-dependent pathway (.Harris et al. 2016). Studies have reported SYT4 in various physiological processes, such as insulin secretion, melanocytic dendrite extension, and exocytosis in mature inner hair cells (IHCs) (.Huang et al. 2018, Johnson et al. 2010). Its aberrant expression is associated with oncogenic activity in diverse cancers, including its role in activating B16-F10 melanocytes by modulating Ca^2+^ influx (.Jia et al. 2020). Moreover, bioinformatics analysis in GC showed that upregulation of SYT4 is positively associated with poorer prognoses (.Yang et al. 2021). However, the specific biological functions and mechanisms of SYT4 in GC remain largely unexplored. Hence, our research aims to elucidate the function of SYT4 and its molecular mechanisms in GC, with a particular focus on its interaction with Ca^2+^ signaling.

The majority of individuals diagnosed with gastric cancer are older, emphasizing the urgent need for advancements in treatment. Recent research underscores the importance of ion channels, especially those involved in Ca^2+^ signaling, as novel therapeutic targets in cancer treatment. Given the crucial role of Ca^2+^ signaling in various cell death pathways, further investigation into its function in the progression of gastric cancer is vital. By delving into the function of SYT4 and its molecular mechanisms in GC, particularly its interaction with Ca^2+^ signaling, this study could uncover new therapeutic strategies, providing more effective treatment options for patients with gastric cancer.

## Methods and materials

### Bioinformatic analysis

mRNA expression profiles were extracted from The Cancer Genome Atlas (TCGA) and the Gene Expression Omnibus (GEO) database, specifically series number GSE56807. Data were processed using GeneBASE software. Using RMA to get the gene core signal from each probe. The expression value of SYT represents the normalized value of Affymetrix gene chip. The Genotype-Tissue Expression (GTEx) database was used to obtain the normal tissues for Receiver Operating Characteristic (ROC) analysis. Kaplan-Meier curves analyzed overall survival (OS), while Receiver Operating Characteristic (ROC) analysis assessed diagnostic performance. The surv_cutpoint function was utilized to identify the optimal cutoff of SYT4 for the dataset. The maximum statistic was set as the best cutpoint. Patients were stratified into high and low SYT4 expression groups based on the cutoff value, and differentially expressed genes (DEGs) were identified by DESeq2 package and visualized by the ggplot2 package. Gene Ontology (GO) biological process enrichment and Kyoto Encyclopedia of Genes and Genomes (KEGG) pathway analyses were conducted via DAVID (https://david.ncifcrf.gov/tools.jsp), presented in enrichment scatter plots. Gene Set Enrichment Analysis (GSEA) further elucidated related molecular signaling pathways.

### Patients and specimens

A retrospective analysis was conducted on 374 primary gastric cancer (GC) cases presented between January 2014 and December 2020 at Zhongshan Hospital, Fudan University, Shanghai, China. Informed consent was comprehensively obtained from all patients involved in the study, which was approved by the Institutional Review Board (IRB) of Zhongshan Hospital, Fudan University.

### Animals

The Animal Ethics Approval for our study was granted by the Institutional Animal Care and Use Committee (IACUC). Our study ensures that all procedures involving animals are performed to minimize suffering and distress. Additionally, our research team is committed to adhering to the principles of the 3Rs (Replacement, Reduction, and Refinement) to ensure ethical treatment and use of animals in research.

### Immunohistochemical staining

The EnVision two-step method conducted immunohistochemical staining.The immunohistochemistry (IHC) analysis was carried out in the platform of Leica (Bond-III). GC tissue microarrays were incubated with primary antibodies overnight at 4 °C, followed by a secondary antibody at room temperature for 20 min. Diaminobenzidine (DAB) visualized the staining. The antibodies used in this IHC experiment were listed as follows: anti-SYT4 (1:600 dilution, Invitrogen PA5-87026), anti-phospho-ERK (1:500 dilution, Proteintech 28733-1-AP), anti-phospho-p38 (1:500 dilution, Proteintech 28796-1-AP), anti-caspase-3 (1:600 dilution, Proteintech 19677-1-AP), anti-Ki67 (1:500 dilution, Proteintech 27309-1-AP).

Slides were assessed for staining intensity (0 to 3) and the percentage of positively stained cells (0-100%). Staining intensity was categorized as: weak positive (light yellow staining, scored as 1), moderate positive (brown-yellow, scored 2), and strong positive (brown, scored 3). The immunohistochemistry result was calculated by H-score =∑ (percentage of positive cells × staining intensity) = (range of weak positive × 1) + (range of moderate positive × 2) + (range of strong positive × 3). Cutoff Values: To define high vs. low SYT4 expression groups, a cutoff value is determined based on the H-score of SYT4 with the three-classification method.

### Cell culture and transfection

Immortalized GC and normal gastric epithelial cell lines, procured from the Cell Bank of the Chinese Academy of Sciences, Shanghai, were cultured in Dulbecco’s Modified Eagle Medium (DMEM) supplemented with 10% fetal bovine serum and antibiotics in a humidified 5% CO2 incubator at 37 °C. Lentiviral vectors harboring SYT4 or control sequences (GeneChem, Shanghai, China) facilitated transfections, following manufacturer instructions. Puromycin was used to select stable transfectants, with shRNA sequences was shRNA1-SYT4: 5-GCTCGACATCTGCCTAAATCT-3, shRNA2-SYT4: 5- GTGAACAAGCTGAGAAATTAT-3.

### Quantitative real-time transcription PCR (qRT-PCR)

Total RNA, isolated using Trizol (Invitrogen, CA, USA), was reverse transcribed using a kit from Takara, Dalian, China. qRT-PCR employed the SYBR Green system, with gene expression levels normalized to β-actin and quantified using the 2^−ΔΔCT method. Primer sequences for SYT4 and β-actin are listed below.

5’-TCAGGACGGGGTGAGTTACTG-3’, SYT4-reverse: 5’-GTCGAGCTTTTAAGACAACCACA-3’, β-actin-forward: 5’-GACCTGACTGACTACCTCATGAAGAT-3’, β-actin-reverse: 5’-GTCACACTTCATGATGGAGTTGAAGG-3’.

### Western blotting (WB)

Protein extraction from cell lysates was performed using RIPA buffer supplemented with protease inhibitors (Beyotime, Shanghai, China). Protein concentrations were quantified utilizing the BCA Protein Assay Kit (Beyotime). Subsequent separation of proteins was carried out via SDS-PAGE and transferred onto PVDF membranes (Millipore, Billerica, MA, USA). Membranes were probed with primary antibodies against SYT4 (1:1000 dilution), ERK (1:2000 dilution), phospho-ERK (1:3000 dilution), p38 (1:2000 dilution), phospho-p38 (1:2000 dilution), JNK (1:1000 dilution, Proteintech 66210-1-lg), phospho-JNK (1:1000 dilution, Proteintech 80024-1-RR), and GAPDH (1:20000 dilution, Proteintech 12935-1-AP) as a loading control. Band intensity was quantified using NIH ImageJ software (version 1.8.0.112). Each assay was performed in triplicate to ensure reproducibility.

### Cell proliferation assay

Cellular proliferation was assessed using the Cell Counting Kit-8 (CCK-8, Dojindo, Kumamoto, Japan). HGC27 and MGC803 cells were plated at a density of 5,000 cells per well in 96-well plates. Following a 24-hour incubation period, 10 µl of CCK-8 solution was dispensed into each well. After a suitable incubation time to allow for color development, absorbance was measured at 450 nm utilizing the Multiskan™ FC Microplate Photometer (Thermo Scientific).

### Apoptosis assay

For apoptosis quantification, cells were seeded in 6-well plates and cultured overnight. Post incubation, they were trypsinized, washed, and resuspended in binding buffer. Staining was conducted with 5 µl of PE Annexin V and 5 µl of 7-amino-actinomycin (7-AAD, BD Biosciences, NY, USA). The cells were then incubated for 20 min in the dark, followed by analysis using the BD FACSAria™ III flow cytometer (BD Biosciences, Franklin Lakes, New Jersey).

### Ca^2+^ measurement

Gastric cancer cells were cultured in 6-well plates 24 h prior to the assay. Cells were washed with Hank’s Balanced Salt Solution containing 20 mM HEPES (HHBS) and then incubated with CalbryteTM 520 AM (AAT Bioquest, USA) at 37 °C for 60 min to label intracellular Ca^2+^. Excess dye was removed by replacing the solution with fresh HHBS. Changes in Ca^2+^ levels were visualized using a laser scanning confocal microscope (FV1000, Olympus, Japan).To explore the change of intracellular Ca^2+^ concentration, Ca^2+^ influx upon stimulation with 1 µl ionomycin (StressMarq Biosciences, Beijing, China) was measured by flow cytometry.

### Xenograft tumor model

NKG mice aged 4–6 weeks (Model Organisms, Shanghai, China) were injected subcutaneously with 1 × 10^7^ SYT4-knockdown (KD) MGC803 cell line or control cells in 200 µl phosphate buffer solution (PBS). Each experimental group comprised five mice. Tumors were measured weekly for four weeks, with volumes calculated using the formula: 0.5×width^2^×length. Upon study completion, mice were euthanized. The mice were placed in a sealed experimental animal chamber SMQ-1 (Tianhuan Technology Co. Ltd., Shanghai, China) containing carbon dioxide (CO_2_), and CO_2_ is slowly injected until the mice expire. The tumors were excised and weighed. The protocol was approved by the Institutional Animal Care and Use Committee of Fudan University, Zhongshan Hospital.

### Experimental grouping and treatment with test drugs

To assess the impact of calcium inhibition on cellular viability, SYT4-overexpressing (OE) and corresponding negative control (OENC) cells were pre-incubated for 24 h. Subsequently, cells were exposed to graded concentrations of amlodipine (0, 5, 10, and 20 µM) for 24, 48, and 72 h. Cell proliferation rates were quantified using the CCK8, consistent with prior methodology. Apoptotic responses to calcium inhibition were analyzed in both cell lines using flow cytometry after a 24-hour incubation with 10 µM amlodipine, with dimethyl sulfoxide (DMSO) serving as the control.

### Amlodipine treatment of subcutaneous tumors in mice

In vivo efficacy of amlodipine was tested on NKG mice (4–6 weeks old), which were subcutaneously inoculated with 1 × 10^7^ MGC803-SYT4-OENC or MGC803-SYT4-OE cells (5 mice in each group). Following a 10-day period, allowing tumors to reach approximately 100 mm^3^, mice were administered DMSO (controls) or amlodipine (10 mg/kg/day, orally). Treatment continued for 28 days, after which animals were euthanized, and tumor burdens were evaluated.

### Statistical analysis

Data analysis was conducted using GraphPad Prism 9 and R programming language. Survival outcomes were analyzed via Cox regression, while log-rank tests compared clinicopathological characteristics across patient cohorts. All experiments were replicated three times to ensure reliability.

## Results

### Survival analysis of SYT4 on bioinformatics data

**Fig. 1:**
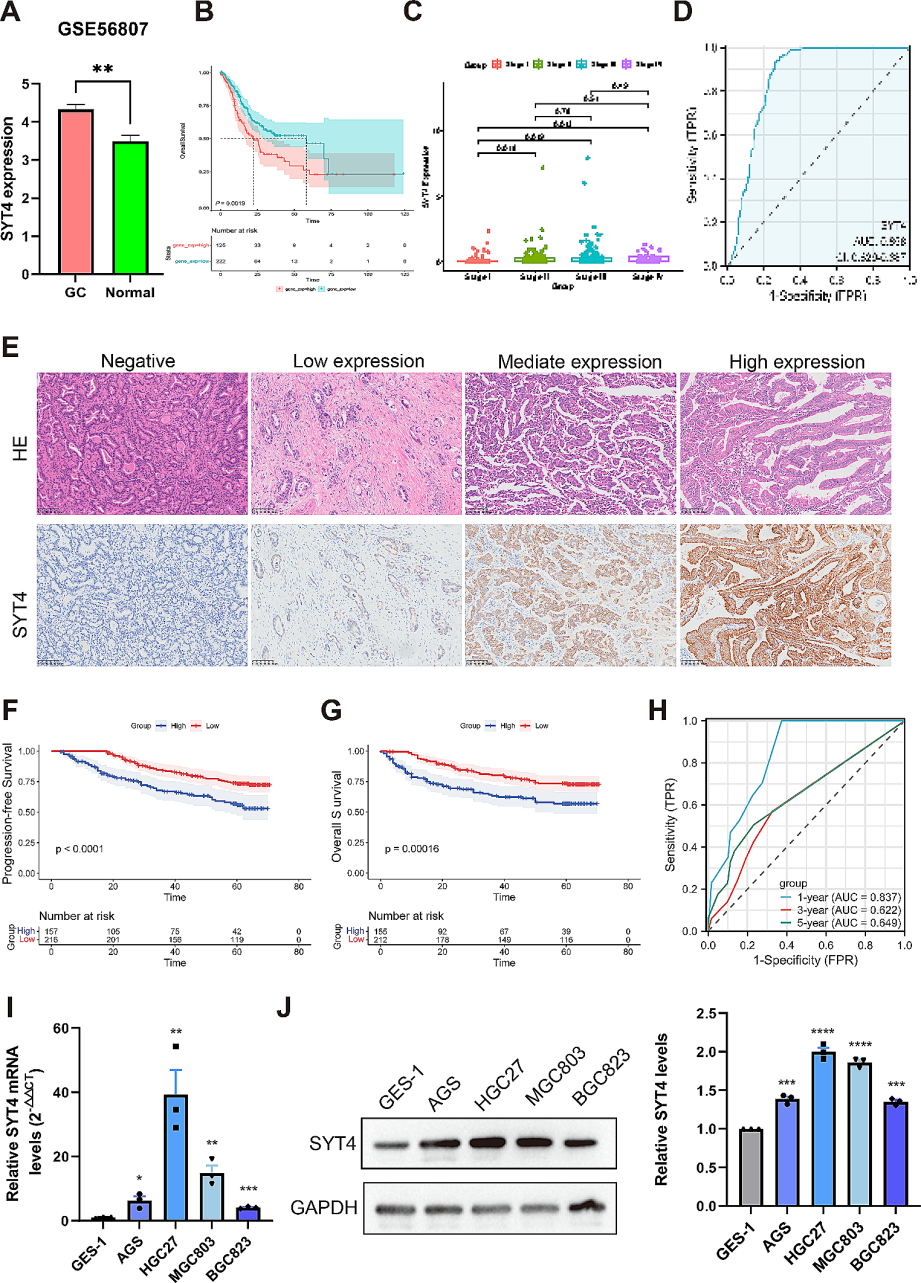
Bioinformatics Analysis and prognostic value of SYT4 in GC. **A** SYT4 mRNA level of GC tissues (*n* = 4) *versus* normal (*n* = 4) from GSE56807 database. **B** The correlation between SYT4 expression and the prognosis of GC patients was performed using bioinformatics. **C** Compared to the early-stage GC, SYT4 expression was notably elevated in advanced-stage GC. **D** ROC curve showed SYT4 was a marker to distinguish GC tissues from normal tissues (AUC value 0.858). **E** Representative images of immunohistochemistry (IHC) staining for SYT4 with different staining intensity. **F and G** Progression-free survival (PFS) and overall survival (OS) analysis of GC patients with high or low SYT4 expression. **H** ROC curve showed that SYT4 could be an effective prognostic marker for the 1- year survival rate (AUC value 0.837), and a suboptimal marker for the 3- and 5- year survival rate in GC patients (AUC value 0.622 and 0.649). **I** The mRNA levels of SYT4 in different cells were measured by qRT-PCR. **J** The expressions of SYT4 in different cells were detected by WB.

GEO and The Cancer Genome Atlas-Stomach Adenocarcinoma (TCGA-STAD) datasets were scrutinized to evaluate SYT4 mRNA levels. According to the previous study (.Yang, Long, Hu, Bin, Chen, Wu, Peng, Wang, Yao and Li 2021), the SYT4 level was up-regulated in GC tissues, based on TGCA database. In GC samples GEO database, SYT4 expression was significantly elevated compared to normal gastric tissue (*P* = 0.0065, Fig. 1A). Kaplan-Meier survival analysis revealed that high SYT4 expression was associated with reduced OS (*P* = 0.0019, Fig. 1B). In advanced-stage GC (stage II- IV), SYT4 exhibited markedly higher expression levels compared to early-stage GC (stage I) (Fig. 1C). ROC curve analysis suggested that SYT4 possesses potential as a diagnostic biomarker for GC, with considerable sensitivity and specificity (Fig. 1D). These findings collectively imply that SYT4 may function as an oncogene and serve as a prognostic indicator in GC.

### Correlation between SYT4 expression and clinicopathological features

Immunohistochemistry (IHC) was conducted on 374 GC and 373 adjacent normal tissue samples. Three-classification method was used based on the H-score of SYT4 to categorize the patients into high or low SYT4 expression groups. The score of 0 (the median) was labeled as “None”, scores ranging from 0 to 20 (the third quartile) were labeled as “Low”, and scores > 20 were labeled as “High”. Notably, GC tissues exhibited a higher incidence of elevated SYT4 expression (198/374) relative to normal tissues (57/373, *P* < 0.05). Positive SYT4 expression was inversely related to both progression-free survival (PFS) and OS (*P* < 0.0001 and *P* = 0.00016, respectively; Fig. 1F-G). Additionally, ROC analysis demonstrated that SYT4 was an effective prognostic marker for the 1- year survival rate (AUC value 0.837), and a suboptimal marker for the 3- and 5- year survival rate in GC patients (AUC value 0.622 and 0.649) (Fig. 1H).

Clinicopathological analysis revealed significant differences in tumor size, vascular invasion and TNM stage (Table 1). Univariate analysis confirmed that age, tumor size, T stage, N stage, lymph node metastasis, vascular invasion, nerve invasion, TNM stage and SYT4 expression were significantly associated with poorer OS (all *P* < 0.001). Multivariate analysis further identified age, T stage, and SYT4 expression as independent prognostic factors for adverse outcomes in GC (Table 2).

**Table 1:** Correlation of clinicopathological features of SYT4 in 374 patients

Characteristics	SYT4-positive	SYT4-negative	*P* value
n	198	176	
Age, n (%)			0.217
≥ 60	126 (33.7%)	101 (27%)	
< 60	72 (19.3%)	75 (20.1%)	
Sex, n (%)			< 0.001
Male	80 (21.4%)	110 (29.4%)	
Female	118 (31.6%)	66 (17.6%)	
Tumor Size, n (%)			0.001
< 5	93 (25%)	112 (30.1%)	
≥ 5	104 (28%)	63 (16.9%)	
Differentiation, n (%)			0.432
Poor	183 (49.2%)	166 (44.6%)	
Well	14 (3.8%)	9 (2.4%)	
T State, n (%)			0.501
T1-2	60 (16.1%)	59 (15.9%)	
T3-4	137 (36.8%)	116 (31.2%)	
N State, n (%)			0.185
0–1	90 (24.2%)	92 (24.7%)	
2–3	107 (28.8%)	83 (22.3%)	
Lymph node metastasis, n (%)			0.278
No	54 (14.5%)	57 (15.3%)	
Yes	143 (38.4%)	118 (31.7%)	
Vascular invasion, n (%)			0.001
No	81 (21.8%)	101 (27.2%)	
Yes	116 (31.2%)	74 (19.9%)	
Nerve invasion, n (%)			0.508
No	70 (18.8%)	68 (18.3%)	
Yes	127 (34.1%)	107 (28.8%)	
TNM State, n (%)			< 0.001
I-II	75 (20.2%)	148 (39.8%)	
III-IV	122 (32.8%)	27 (7.3%)	
Her2, n (%)			0.970
Negative	56 (15%)	51 (13.7%)	
Low-expression	128 (34.3%)	111 (29.8%)	
Amplification	14 (3.8%)	13 (3.5%)	

**Table 2:** Univariate and multivariate analysis of the association of prognosis with clinicopathologic parameters and expression of SYT4 in GC.

Characteristics	Total(N)	Univariate analysis			Multivariate analysis	
		Hazard ratio (95% CI)	*P* value		Hazard ratio (95% CI)	*P* value
Age	373					
≥ 60	227					
< 60	146	0.396 (0.254–0.615)	**< 0.001**		0.520 (0.331–0.816)	**0.004**
Sex	373					
Male	189					
Female	184	1.099 (0.754–1.601)	0.623			
Tumor Size	371					
< 5	205					
≥ 5	166	2.524 (1.707–3.733)	**< 0.001**		1.213 (0.806–1.827)	0.354
Differentiation	371					
Poor	348	Reference				
Well	23	0.603 (0.148–2.452)	0.479			
T State	371					
T1-2	119					
T3-4	252	0.068 (0.025–0.184)	**< 0.001**		0.208 (0.069–0.628)	**0.005**
N State	371					
0–1	182					
2–3	189	5.311 (3.235–8.719)	**< 0.001**		1.752 (0.874–3.515)	0.114
Lymph node metastasis	371					
No	111					
Yes	260	5.999 (2.919–12.328)	**< 0.001**		1.406 (0.545–3.629)	0.481
Vascular invasion	371					
No	182					
Yes	189	0.306 (0.202–0.466)	**< 0.001**		0.826 (0.520–1.313)	0.420
Nerve invasion	371					
No	138					
Yes	233	0.163 (0.087–0.304)	**< 0.001**		0.533 (0.264–1.074)	0.078
TNM State	371					
I-II	223					
III-IV	148	3.896 (2.602–5.835)	**< 0.001**		1.173 (0.623–2.209)	0.622
Her2	372					
Negative	107					
Low-expression	238	1.392 (0.878–2.208)	0.160			
Amplification	27	1.762 (0.863–3.598)	0.120			
SYT4	373					
Positive	197					
Negative	176	0.431 (0.290–0.640)	**< 0.001**		0.546 (0.310–0.960)	**0.036**

### SYT4 promotes GC cell proliferation, inhibits cell apoptosis both in vivo and in vitro

Correlations between SYT4 expression and clinicopathological characteristics of GC prompted investigation into its potential oncogenic role. Elevated SYT4 expression was confirmed in GC cell lines compared to GES-1 cells, at both mRNA and protein levels (Fig. 1I-J). Following transfection with SYT4-specific shRNA (shSYT4-2), which showed the greatest knockdown efficiency in HGC27 and MGC803 cells (Supplementary Fig. S1), further functional assays were conducted.

**Fig. 2:**
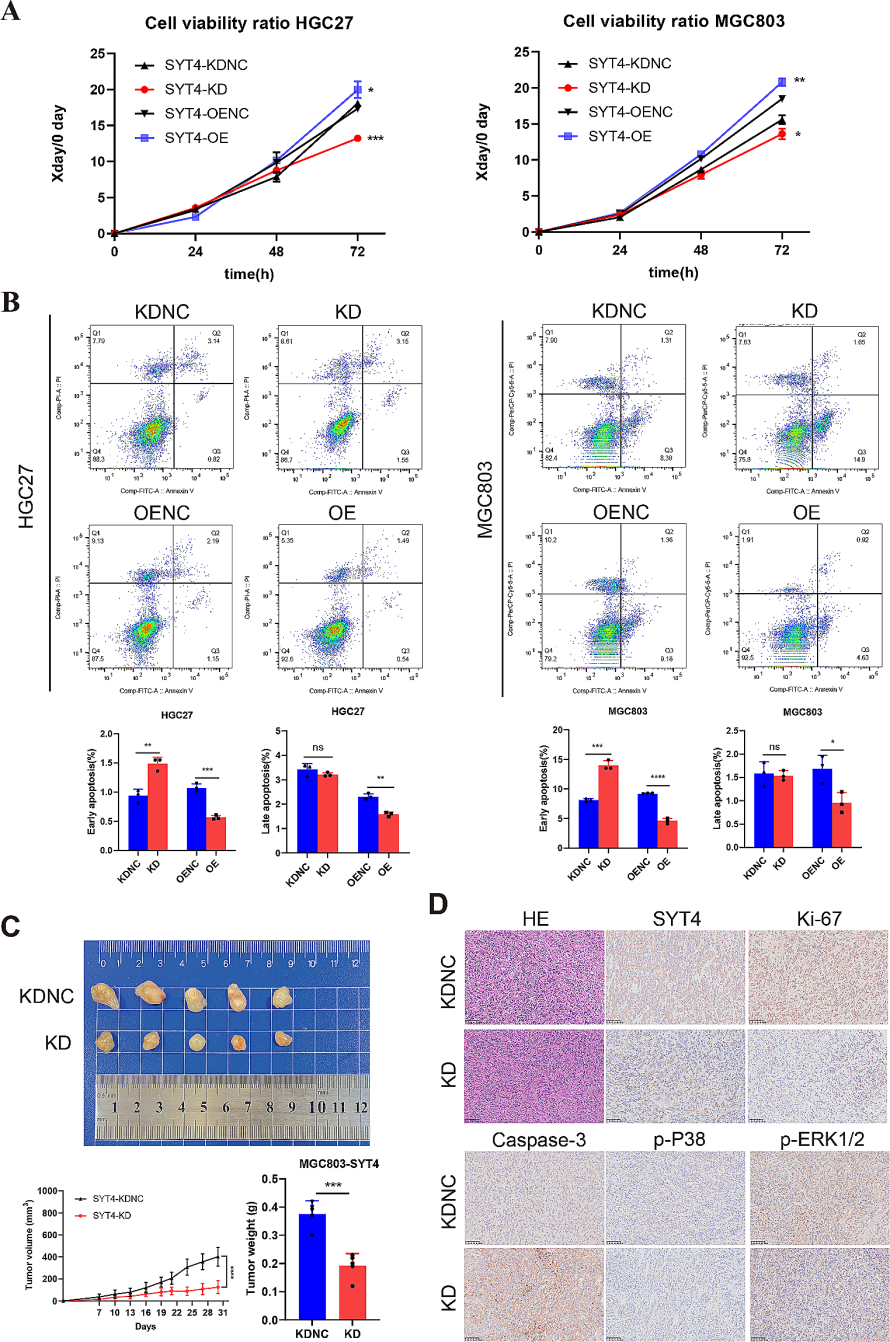
The effects of SYT4 on GC progression in vivo and in vitro. **A** SYT4 knockdown weakened cell viability, while SYT4 upregulation promoted cell viability in HGC27 and MGC803 cells. **B** Downregulation of SYT4 induced cell apoptosis. Conversely, SYT4 overexpression decreased cell apoptosis rate. **C** The growth of GC xenografts in SYT4-KDNC and SYT4-KD groups, and the corresponding growth curves. **D** IHC staining of SYT4, Ki-67, Caspase-3, p-P38 and p-ERK in xenografts

CCK8 assays revealed that SYT4 suppression reduced GC cell proliferation rates, while SYT4 overexpression had the inverse effect (Fig. 2A). Apoptosis assays demonstrated that SYT4 knockdown significantly increased early apoptosis in HGC27 and MGC803 cells compared to controls (*P* < 0.01). Conversely, SYT4 upregulation greatly impeded cell apoptosis rate *(P* < 0.001 and *P* < 0.05, Fig. 2B). In vivo validation using NKG mice inoculated with SYT4-KD cells produced tumors with reduced volume and weight, corroborating the in vitro findings (Fig. 2C). Immunohistochemical staining of tumor sections showed decreased expression of SYT4, Ki-67, and increased Caspase-3 in the knockdown group compared to controls (Fig. 2D). Collectively, these findings suggest that SYT4 significantly promotes cell proliferation, hinders apoptosis in vivo, and contributes to tumor growth in a mouse model.

### The effect of SYT4 on intracellular Ca^2+^

**Fig. 3:**
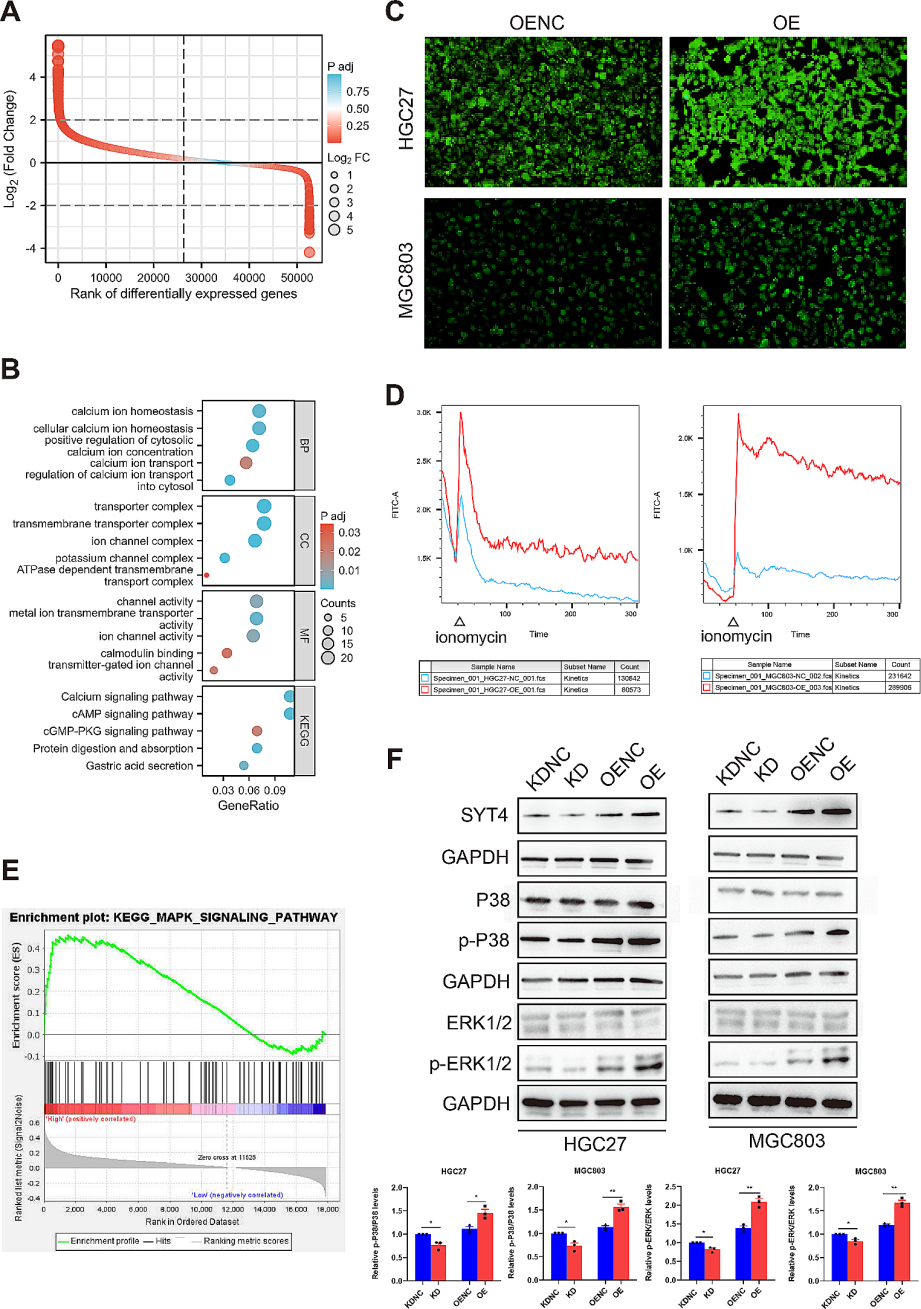
Effect of the SYT4 on calcium influx and MAPK signaling pathway. **A** and **B** The difference analysis and GO/KEGG analysis were visualized by ggplot2 package. Bubble chart showed the enrichment of calcium ion related functions. **C** and **D** Overexpression of SYT4 in GC cells promoted calcium influx showed by confocal laser scanning and flow cytometry analysis. **E** The potential downstream pathway (MAPK) of SYT4 was detected by GSEA, with the normalized enrichment score (NES) of 1.68 and false discovery rate (FDR) of 0.18. **F** The results from WB and abundance analysis of MAPK-related protein (p-P38 and p-ERK1/2) expression levels

GO and Kyoto Encyclopedia of Genes and Genomes (KEGG) analyses of differentially expressed genes highlighted significant enrichment in calcium ion-related functions (Fig. 3A-B). Previous studies have implicated SYT4 involved in cancer progression via modulation of Ca^2+^ influx. We examined the impact of SYT4 on intracellular Ca^2+^ levels in GC cells. Ca^2+^ imaging revealed that the SYT4-OE group exhibited a markedly stronger fluorescence signal compared to the negative control SYT4-OENC group (Fig. 3C). Flow cytometry analysis confirmed that upon stimulation with ionomycin, the SYT4-OE group experienced a pronounced increase in intracellular Ca^2+^, which stabilized over time (Fig. 3D). These findings suggest that SYT4 overexpression may elevate intracellular Ca^2+^ levels.

### SYT4 activates MAPK signaling pathway

Further KEGG enrichment analysis connected SYT4 with the MAPK signaling pathway (Fig. 3E). WB assays demonstrated that SYT4 knockdown resulted in a substantial decrease in phosphorylated ERK 1/2 (p-ERK1/2) and p-P38 levels, while SYT4 overexpression produced the opposite effect. Similar trends could be observed in the xenograft tumor tissues (Fig. 2D). These observations indicate that SYT4 could be involved in the regulation of GC proliferation and apoptosis through the activation of the MAPK pathway.

### Modulation of GC proliferation by Ca^2+^ channel inhibition

**Fig. 4:**
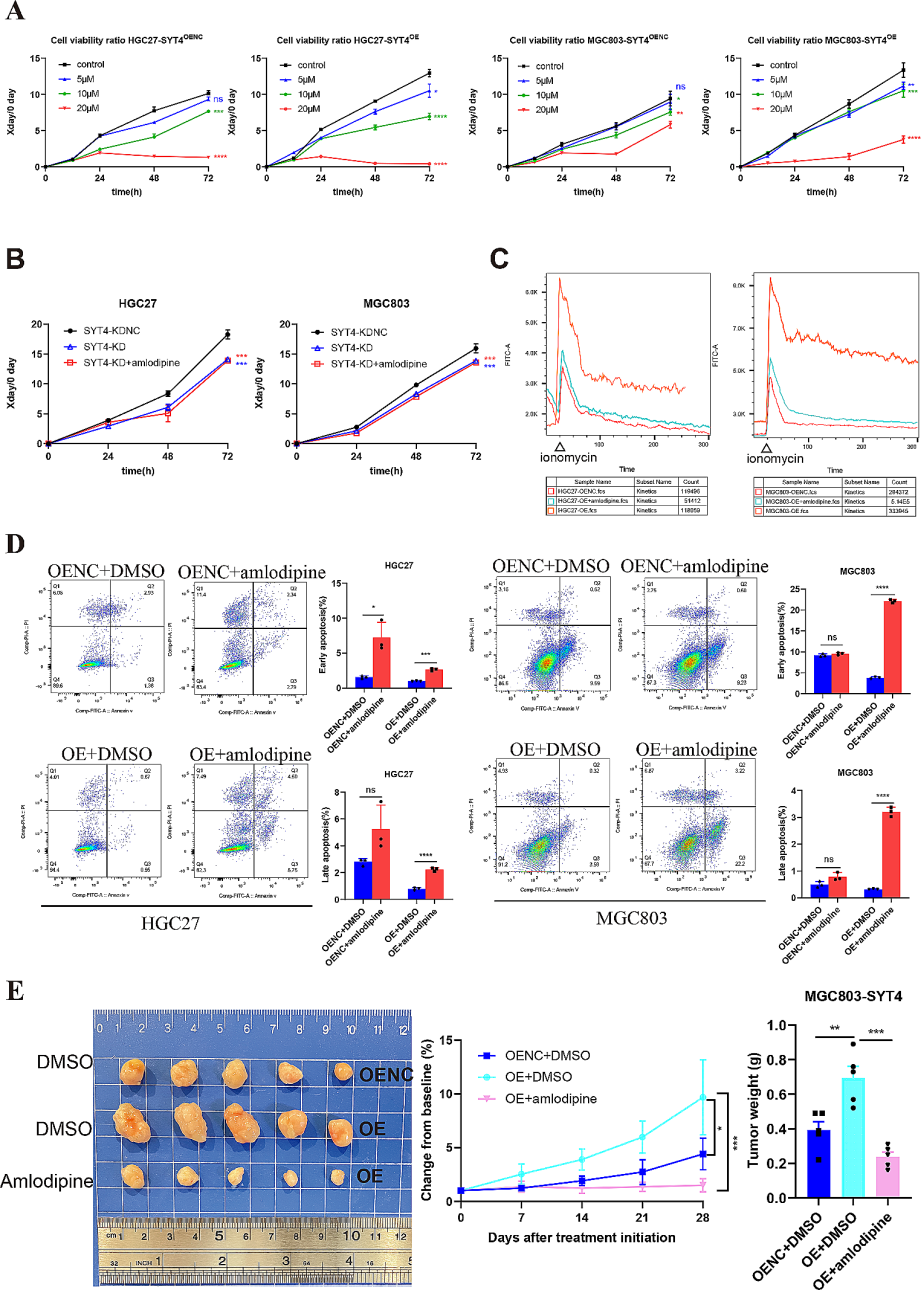
Blockade of Ca^2+^ channel by Ca^2+^ channel amlodipine impaired SYT4-induced tumorigenesis in GC. **A** Amlodipine showed an inhibition effect on GC cell lines in a concentration-dependent manner. The prohibition effect was more severe in SYT4-OE group. **B** Amlodipine has no significant inhibitory effect on SYT4-KD cells. **C** The heightened intracellular Ca^2+^ level induced by SYT4 overexpression would be reversed by amlodipine treatment. **D** For cell apoptosis analysis amlodipine had little effects on the control group but promoted apoptosis in the SYT4-OE group, reaffirming the inhibitory effect of amlodipine on GC cell apoptosis was partly dependent on SYT4. **E** The anticancer effect of amlodipine in vitro. The treatment of amlodipine significantly decreased mean tumor volume and weight in SYT4 overexpression mice compared to the DMSO group

Since SYT4 exerts its function depending on calcium (.Bilbao et al. 2008) and may participate in the regulation of Ca^2+^ homeostasis in GC, we investigated the impact of Ca^2+^ channel inhibition on SYT4-mediated GC progression. Amlodipine was administered at concentrations of 0, 5, 10, or 20 µM to GC cell lines. CCK8 assay indicated that amlodipine inhibited cell proliferation dose-dependently in both the control and SYT4-OE cells, with a more pronounced effect observed in the SYT4-OE group (Fig. 4A). We selected the lowest effective concentration 10 µM for further experimentation.

To further investigate whether amlodipine could inhibit the SYT4-related calcium channels. SYT4-KD cells were treated with amlodipine and the result showed the inhibition of cell proliferation in both the SYT4-KD group and the SYT4-KD + amlodipine group compared to the control group. However, there was no significant difference in the inhibition between these two groups (Fig. 4B), indicating that inhibitory effect of amlodipine on cell proliferation was notably weakened after SYT4 knockdown. This finding suggested that the impact of amlodipine on GC proliferation inhibition was partly dependent on SYT4. Besides, as shown in Fig. 4C, the heightened intracellular Ca^2+^ level induced by SYT4 overexpression would be reversed by amlodipine treatment, suggesting a suppressive effect of amlodipine on SYT4 to some extent. Subsequently, apoptosis assays were conducted with 10 µM amlodipine over 24 h. Apoptosis was significantly increased in the SYT4-OE cells relative to controls (Fig. 4D).

**Fig. 5:**
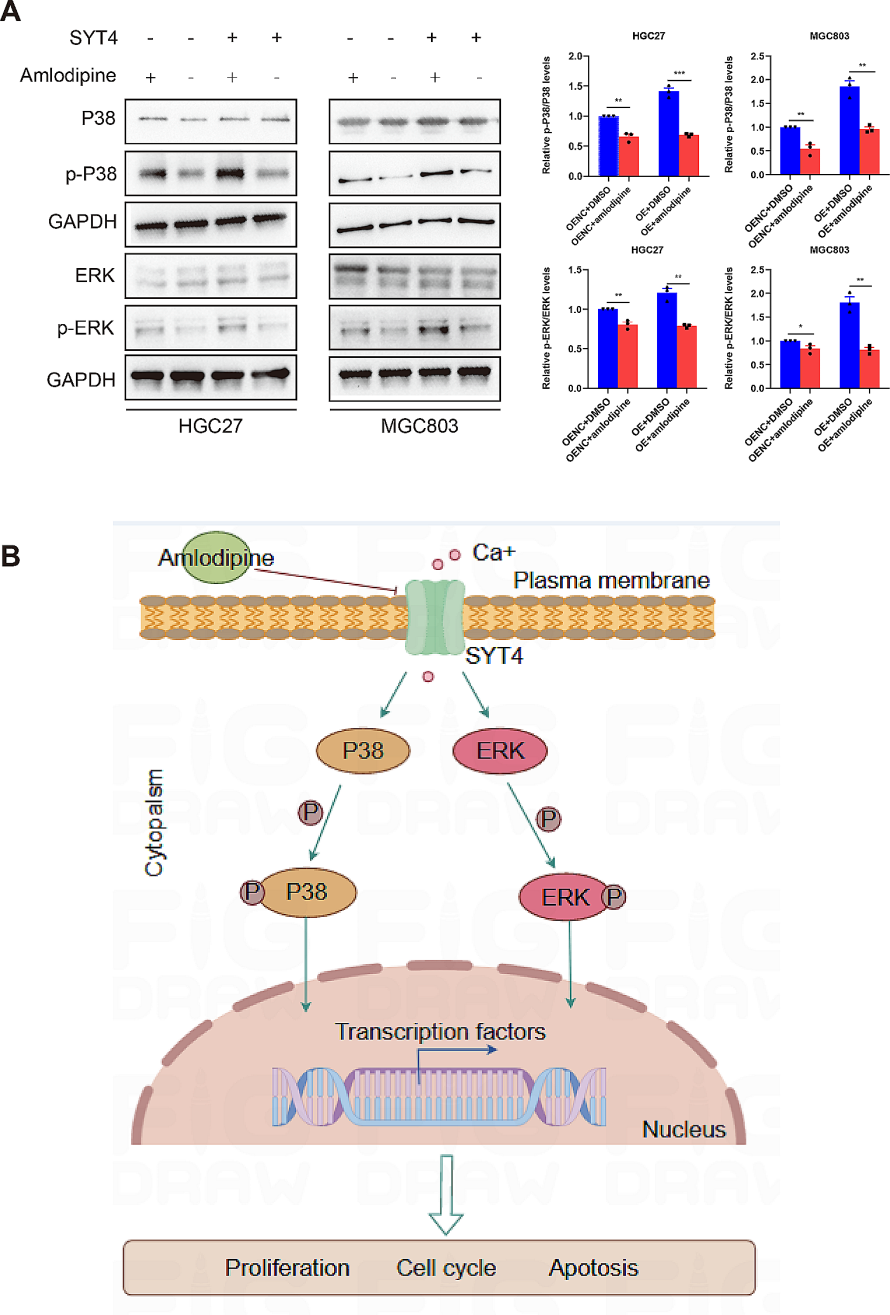
**A** Expression of MAPK-related proteins in SYT4-overexpressed and control GC cells were detected using western blotting after amlodipine treatment. The activation of MAPK signaling caused by SYT4 upregulation could be attenuated by the Ca^2+^ inhibitor. **B** Graphic showing the signaling pathway by which SYT4 regulates GC via Ca^2+^ influx

In vitro, the anti-proliferative efficacy of amlodipine was validated in NKG mice inoculated with SYT4-OE and control cells. Oral administration of amlodipine substantially reduced tumor growth and volume compared to the DMSO-treated group in SYT4-OE group (Fig. 4E). Further investigations into the MAPK pathway revealed that amlodipine treatment resulted in a significant reduction of p-P38/P38 and pERK/ERK ratios in both SYT4-OE and control groups, suggesting a dampening effect on the SYT4-mediated MAPK pathway (Fig. 5A). These results proposed that Ca^2+^ channel blockade may attenuate SYT4-driven GC proliferation both in vivo and in vitro by downregulating the MAPK signaling pathway (Fig. 5B).

## Discussion

GC is the world’s fourth most prevalent malignancy, responsible for nearly one million deaths annually (.Morgan et al. 2022, Song et al. 2022). Despite the widespread adoption of surgical resection, chemoradiotherapy, and targeted therapies, their effectiveness is often undermined by late-stage diagnosis and the development of chemoresistance (.Wang et al. 2020). This underscores the critical need to elucidate the underlying mechanisms of GC to improve early detection and prevention strategies. Recent evidence has implicated synaptotagmin-4 (SYT4) in a broad spectrum of physiological and pathological processes (.Berton et al. 2000, Jia et al. 2020, Patel et al. 2021). However, the role of SYT4 in GC remained elusive until this investigation. Our study provided evidence that SYT4 was upregulated in GC at both the mRNA and protein levels. Notably, elevated SYT4 expression was correlated with aggressive clinical features and poor patient outcomes. Univariate and multivariate analyses suggested that the expression of SYT4 was an independent factor for GC. These findings propose SYT4 as a potential biomarker for prognostication in GC patients.

As a member of the synaptotagmin family, SYT4 is known to modulate membrane trafficking and act as a calcium signal sensor (.Bharat et al. 2017, Delignat-Lavaud et al. 2022). It has been documented to regulate insulin granule positioning and pancreatic β-cell maturation (.Blum et al. 2012). Previous study reported that the aberrant expression of SYT4 was associated with melanogenesis and dendrite extension B16-F10 cells (.Jia et al. 2020). Based on the bioinformatics analysis, the expressions of SYT4, SYT9, and SYT14 were up-regulated in GC and contributed to an unsatisfactory OS and PFS in GC patients (.Yang, Long, Hu, Bin, Chen, Wu, Peng, Wang, Yao and Li 2021). Followed by LASSO regression analysis and the Venn diagram, eight co-expression genes (SYT4, ENPP6, VMP1, LY6E, SHISA6, TMEM158, IL11, and KLK8) were discovered that identified some hub genes could be useful in predicting prognosis and management in GC (.Wu et al. 2023). However, the role of SYT4 in GC remains dimness. In this study, through the GEO database SYT4 was upregulated in GC tissues and the its elevated level was closely associated with a worse OS, in line with previous findings. Moreover, tissue microarray results reveal that high expression of SYT4 is closely associated with poorer clinical pathological features and an unfavorable prognosis in GC patients. Subsequently, depletion of SYT4 suppressed GC cell proliferation and enhanced cell apoptosis, while the inverse results were observed after SYT4 overexpression. SYT4 knockdown impeded GC growth in murine models, echoing the in vivo results. Collectively, these findings support the hypothesis that SYT4 acts as an oncogenic driver in GC pathogenesis.

The role of intracellular Ca^2+^ concentration in regulating cellular processes in GC has been intensively explored. Proteins like S100A14 have been shown to inhibit metastasis by curtailing store-operated Ca^2+^ entry (.Zhu et al. 2017), while ORAI2 expression has been linked to GC prognosis and tumorigenicity through Ca^2+^ signaling modulation (.Kokoska, Smith, Wolff, Deshpande, Rieckenberg, Banan and Miller 1998, Wu et al. 2021). Given that Ca^2+^ acts as a ubiquitous intracellular messenger (.Panneerpandian et al. 2021, Zhang et al. 2020), and the synaptotagmin (SYT) family’s role as Ca^2+^ sensors, Jia et al. discovered that SYT4 was a key factor in promoting melanogenesis through dendrite extension and tyrosinase activity by the regulation of Ca^2+^ influx (.Jia et al. 2020). Our study delved into SYT4’s role in GC Ca^2+^ dynamics and obtained results similar to those of earlier study. GO/KEGG analyses revealed significant enrichment in calcium-related functions, and Ca^2+^ imaging illustrated enhanced influx in SYT4-overexpressing GC cells. Ionomycin treatment further amplified intracellular Ca^2+^ levels in these cells, suggesting that SYT4 upregulation increases free intracellular Ca^2+^ in GC.

The investigation into SYT4’s oncogenic mechanism highlighted the MAPK signaling pathway as a key player, known for its role in extracellular signal mediation (.Sun et al. 2019). Phosphorylation of MAPK components is recognized as crucial for cell proliferation, migration, apoptosis, and differentiation (.Li et al. 2022, Xu et al. 2020). For example, SLC39A10 has been reported to drive malignancy via MAPK/ERK signaling (.Ren et al. 2023). P38 MAPK plays dual roles in regulating tumor cell growth, mediating survival and cell death signals at the same time (.Jin et al. 2016). Liu et al. demonstrated the protective role of P38 MAPK in the initiation of cancer (.Liu et al. 2007). However, tumors with high tumorigenicity were unable to respond to the apoptotic effects caused by p38 MAPK activation following ROS generation (.Park et al. 1999). These studies suggested that the ultimate function of p38 MAPK activation mainly depended on the type of stimulus in a cell-type-specific manner (.Jin, Mo, Zhang, Gao, Wu, Li, Hao, Ma, Gao and Chen 2016). In this study, we found that p-ERK1/2 and p-P38 level were significantly enhanced in SYT4-transfected cells, while deletion of SYT4 induced the protein level reduction. The above findings indicated that SYT4 contributed to the malignant biology of GC by activating the ERK and p38 MAPK pathway, which deserves further studies.

A pivotal aspect of our study was discerning the role of SYT4 in GC progression through a Ca^2+^-dependent pathway. Utilizing amlodipine, a Ca^2+^ channel blocker known for its cardiovascular applications (.Taylor and Simpson 1992), we investigated SYT4’s influence on Ca^2+^ activity within tumorigenesis. Previous research has established amlodipine’s antineoplastic properties across several cancer types, including GC (.Wei et al. 2007), hepatocellular (.Shiozaki et al. 2023) and breast cancers (.Alqudah et al. 2022). Shiozaki et al. demonstrated the effectiveness of amlodipine in curtailing the proliferation of GC stem cells, and the combination of cisplatin and amlodipine impaired tumorigenesis ability in mice (.Shiozaki, Katsurahara, Kudou, Shimizu, Kosuga, Ito, Arita, Konishi, Komatsu, Kubota, Fujiwara, Okamoto and Otsuji 2021). Our data are in agreement, illustrating that amlodipine diminishes SYT4-mediated enhancement of GC cell proliferation, corroborated by both in vivo and in vitro models. Flow cytometry analysis suggested that the apoptosis induced by SYT4 overexpression is potentially Ca^2+^-independent, as Ca^2+^ suppression via amlodipine led to increased apoptotic rates in SYT4-OE cells. Moreover, amlodipine treatment resulted in a notable reduction of phosphorylated P38 and ERK levels in the SYT4-OE cohort, signifying the inhibition of MAPK pathway activation. These observations suppose that while SYT4’s effect on GC progression is mediated through MAPK signaling, its regulation of intracellular Ca^2+^ is instrumental in this process.

The discovery that elevated SYT4 expression in GC tissues is significantly correlated with a worse prognosis positions SYT4 as a promising prognostic biomarker. This insight allows for the stratification of GC patients based on SYT4 expression levels, potentially guiding treatment decisions and follow-up strategies. Moreover, the fact that SYT4 promotes tumorigenesis through mechanisms such as cellular proliferation, inhibition of apoptosis, and enhancement of intracellular Ca^2+^ influx, especially via MAPK pathway activation, underlines its potential as a therapeutic target. While the study presents compelling evidence of SYT4’s role in GC, it is not without limitations. The study mainly focuses on bioinformatics analyses, immunohistochemistry, and in vitro and in vivo functional assays to delineate SYT4’s role. However, the molecular mechanisms underlying SYT4’s effects on GC progression, particularly its interaction with the MAPK pathway and other potential signaling pathways, require further elucidation. Additionally, the study’s reliance on a calcium channel blocker for therapeutic intervention needs exploration in the context of specificity and side effects when applied to GC treatment.

## Conclusion

This study reveals SYT4’s overexpression in gastric cancer (GC) links to poor prognosis and aggressive traits, indicating its role in GC progression. SYT4 boosts cell growth and blocks apoptosis via Ca^2+^-MAPK pathways. Amlodipine’s effectiveness against SYT4’s oncogenic effects suggests calcium channel modulation as a viable therapeutic strategy in GC, positioning SYT4 as a key biomarker and therapeutic target.

### Electronic supplementary material

Below is the link to the electronic supplementary material.

**Supplementary Figure S1**: The transduction efficiency of SYT4 in HGC27 and MGC803 cells detected by qRT-PCR and WB.

## Data Availability

The raw data have been uploaded to the Figshare database, Hou, Yingyong (2024). raw data. figshare. collection. https://doi.org/10.6084/m9.figshare.c.7024320.v1.
