# Supplementary figures and images for: Amlodipine inhibits Synaptotagmin-4’s oncogenic activity on gastric cancer proliferation by targeting calcium signaling
Source: Funct Integr Genomics. 2024 Apr 18;24(3):77. doi: 10.1007/s10142-024-01345-8 (PMC11024009; doi:10.1007/s10142-024-01345-8)

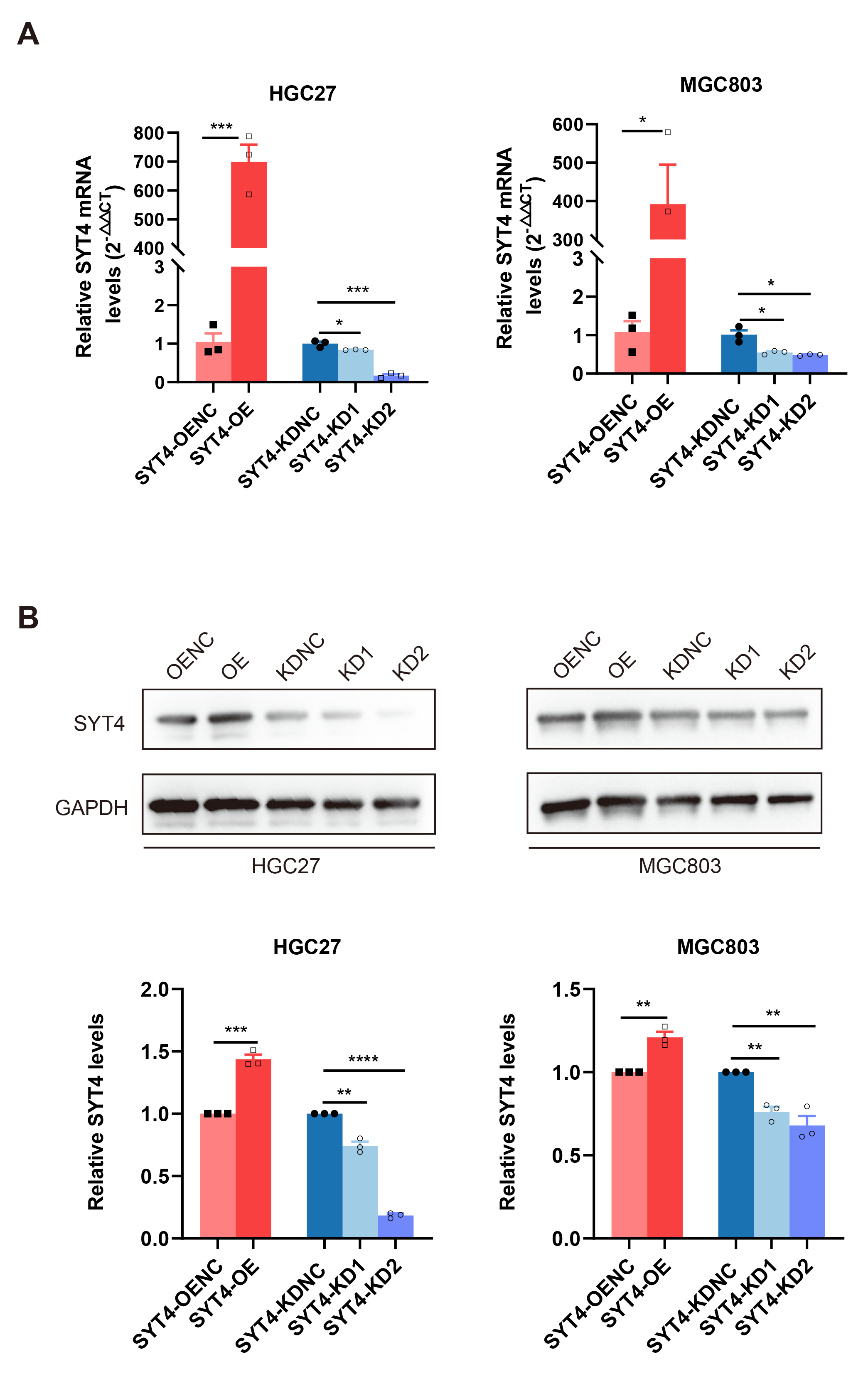

Supplement: Supplementary file 1 — Supplementary Figure S1: The transduction efficiency of SYT4 in HGC27 and MGC803 cells detected by qRT-PCR and WB. [file 10142_2024_1345_MOESM1_ESM.tif]
